# Voxel-Based Morphometry of Cerebellar Lobules in Essential Tremor

**DOI:** 10.3389/fnagi.2021.667854

**Published:** 2021-06-10

**Authors:** Richard Ågren, Amar Awad, Patric Blomstedt, Anders Fytagoridis

**Affiliations:** ^1^Department of Neuroscience, Karolinska Institutet, Stockholm, Sweden; ^2^Unit of Functional and Stereotactic Neurosurgery, Department of Pharmacology and Clinical Neuroscience, Umeå University, Umeå, Sweden; ^3^Physiology Section, Department of Integrative Medical Biology, Umeå University, Umeå, Sweden; ^4^Umeå Center for Functional Brain Imaging (UFBI), Umeå University, Umeå, Sweden; ^5^Department of Clinical Neuroscience, Karolinska Institutet, Stockholm, Sweden

**Keywords:** essential tremor, cerebellum, voxel-based morphometry, lobule volume, structural MRI

## Abstract

**Background:**

The extent of neurodegeneration underlying essential tremor (ET) remains a matter of debate. Despite various extents of cerebellar atrophy on structural magnetic resonance imaging (MRI), previous studies have shown substantial heterogeneity and included a limited number of patients. Novel automated pipelines allow detailed segmentation of cerebellar lobules based on structural MRI.

**Objective:**

To compare the volumes of cerebellar lobules in ET patients with those in healthy controls (HCs) using an automated segmentation pipeline.

**Methods:**

Structural MRI scans of ET patients eligible for deep brain stimulation (*n* = 55) and of age-matched and gender-matched HCs (*n* = 55, from the IXI database) were segmented using the automated CEREbellum Segmentation pipeline. Lobule-specific volume differences between the ET and HC groups were evaluated using a general linear model corrected for multiple tests.

**Results:**

Total brain tissue volumes did not differ between the ET and HC groups. ET patients demonstrated reduced volumes of lobules I-II, left Crus II, left VIIB, and an increased volume of right X when compared with the HC group.

**Conclusion:**

A large cohort of ET patients demonstrated subtle signs of decreased cerebellar lobule volumes. These findings oppose the hypothesis of localized atrophy in cerebellar motor areas in ET, but not the possibility of cerebellar pathophysiology in ET. Prospective investigations using alternative neuroimaging modalities may further elucidate the pathophysiology of ET and provide insights into diagnostic and therapeutic approaches.

## Introduction

Essential tremor (ET) is the most prevalent movement disorder, affecting approximately 4% of the population over 40 years of age (Zesiewicz et al., 2010). Although it was initially believed to be a benign motor disorder, reports have demonstrated increased occurrences of mood disorders, cognitive disorders, and early mortality in ET patients (Benito-Leon et al., 2006; Louis et al., 2007a,b). A neurodegenerative origin of ET has been proposed based on neuropathological analyses of ET patients showing cerebellar pathology (Rajput et al., 2012; Benito-León, 2014). Mechanistically, a cerebello-thalamo-cortical network dysfunction has been suggested to cause tremor in ET (Hallett, 2014; Buijink et al., 2015) and lesions or deep brain stimulation (DBS) in this pathway may suppress tremor (Dupuis et al., 2010; Fytagoridis et al., 2012; Elias et al., 2016; Barbe et al., 2018).

Structural magnetic resonance imaging (MRI), which allows for voxel-based morphometric (VBM) analysis, has demonstrated diverse cortical and cerebellar volumetric changes in ET patients. Decreased cerebellar gray matter has been observed in groups of ET patients (Benito-Leon et al., 2009; Bhalsing et al., 2014; Gallea et al., 2015; Dyke et al., 2017). However, other studies have reported no differences (Daniels et al., 2006; Fang et al., 2013; Nicoletti et al., 2015) and abnormalities in white matter (Pietracupa et al., 2019). A recent meta-analysis of 16 structural MRI investigations proposed that previously reported gray matter abnormalities in ET might be related to methodological heterogeneity and small or diverse cohorts, thereby indicating the need for larger studies using standardized protocols (Luo et al., 2019).

In the present study, cerebellar volumes of 55 ET patients eligible for DBS (Fytagoridis et al., 2012) were compared with those of matched healthy controls (HCs) from the IXI repository. We aimed to test the hypothesis of regional atrophy in the cerebellar motor areas of ET patients using an automated segmentation pipeline (Romero et al., 2017).

## Methods

### Patient and Control Selection

Patients diagnosed with ET (*n* = 70) who underwent T1-weighted MRI before DBS between 2006 and 2015 at Umeå University Hospital, Sweden were included. The exclusion criteria were the use of alternative MRI protocols (*n* = 13), previous DBS electrode implantation (*n* = 1), or major brain lesions (*n* = 1). T1-weighted MRI scans of HCs (*n* = 55) were retrieved from the IXI repository, which included volunteers from three UK hospitals (https://brain-development.org/ixi-dataset/). Subject characteristics (age and sex) and MRI data were collected. To reduce the technical bias introduced by different scanners and sites, volumes were normalized to total brain tissue, which did not differ significantly between the HC and ET groups (Table 1 and Supplementary Figure 1).

**TABLE 1:** Subject characteristics and total brain volumes from volBrain.

Group	HC	ET	
Database, country	IXI repository, United Kingdom	Umeå University Hospital, Sweden	
N	55	55	
Male/Female	27/28	29/26	
Age (years)	67.0 (8.4)	67.3 (8.3)	*p* = 0.8372
Total brain (cm^3^)	1130 (118)	1158 (129)	*p* = 0.2403

*Data are shown as means (SD). *Groups were compared using t-tests.**

### MRI Imaging Procedure

ET patients underwent imaging at Umeå University Hospital, Umeå, Sweden. T1-weighted sequences were acquired using the NT Intera 1.5T MRI scanner (Philips, Amsterdam, Netherlands) with repetition time = 25 ms, echo time = 5.5 ms, number of phase encoding steps = 256, echo train length = 1, reconstruction diameter = 260 mm, and flip angle = 25°. The HC group underwent imaging at Guy’s Hospital, UK with a 1.5T scanner (Philips, Amsterdam, Netherlands) with repetition time = 9.813 ms, echo time = 4.603 ms, number of phase encoding steps = 192, echo train length = 0, reconstruction diameter = 240 mm, and flip angle = 8° (https://brain-development.org/ixi-dataset/).

### Volumetric Analysis

**FIGURE 1:**
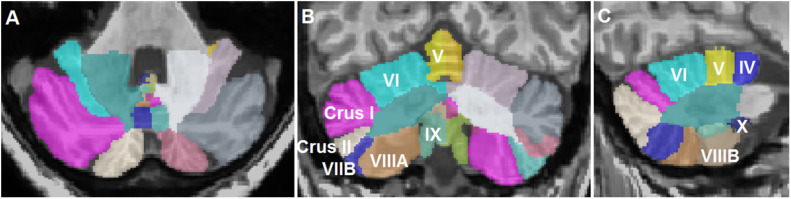
Representative segmentation of a T1-weighted magnetic resonance imaging scan of a patient with essential tremor using the CEREbellum Segmentation pipeline (Romero et al., 2017). **(A)** Axial, **(B)** coronal, and **(C)** sagittal representations of the segmented lobules.

T1-weighted MRI images in DICOM format were anonymized and converted to the NIFTI format using dcm2niix (Li et al., 2016). Cerebellar lobule volumes were determined using the CEREbellum Segmentation (CERES) pipeline, which is a patch-based multi-atlas segmentation pipeline that parcellates the cerebellum into 12 lobules and determines the respective volumes in the native space (Romero et al., 2017). CERES has been demonstrated to outperform several cerebellar pipelines in terms of segmentation accuracy (Romero et al., 2017). Total lobule volumes including both white and gray matter were extracted from the results. The anatomical segmentations in CERES are shown in Figure 1. Total brain volumes were determined by analyzing the T1-weighted sequences using the volBrain pipeline (Manjón and Coupé, 2016).

### Statistical Analysis

Baseline characteristics (age and brain volume) were compared using Student’s *t*-test. Cerebellar regions were compared between the ET and HC groups using a multivariate general linear model with age and total brain volume as covariates. The significance level was adjusted for multiple testing using the Benjamini-Hochberg method (Benjamini and Hochberg, 1995). Statistical analysis was performed using IBM SPSS Statistics version 26 (IBM Corp., Armonk, NY, United States) and GraphPad Prism 8 (GraphPad Software, San Diego, CA, United States).

## Results

**FIGURE 2:**
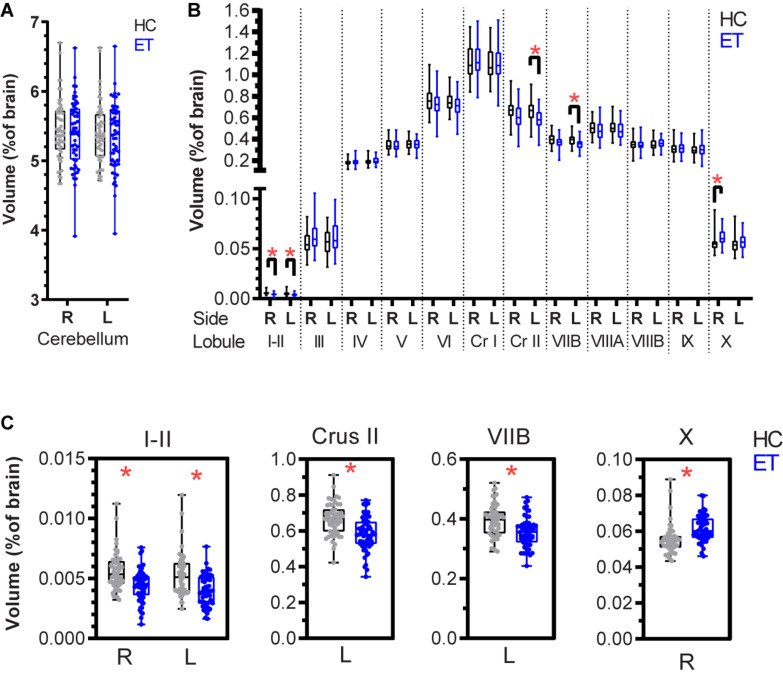
Normalized cerebellar lobule volumes in patients with essential tremor (ET) and in healthy controls (HCs) expressed as percentage of total brain volume. **(A)** Right and left cerebellar lobule volumes for the HC (black) and ET (blue) groups. **(B)** Lobule volumes for the right and left cerebellar hemispheres. **(C)** Lobules with significant volume differences between the HC and ET groups: bilateral I-II, left Crus II, left VIIB, and right X (Table 2). The boxplots show the 25th, 50th, and 75th percentiles for all data. A multivariate general linear model was applied to the cerebellum and the 12 lobules (right and left sides) with gender, age, and total brain volume as covariates. The significance level (*p* < 0.05) was adjusted for multiple testing using the Benjamini-Hochberg method. Significance is denoted by *. R: right, L: left, Cr I: Crus I, Cr II: Crus II.

The ET patients were matched with HCs from the IXI repository based on age and sex (Table 1). The images were matched based on the MRI scanner type and magnetic field strength. Total brain volumes determined using volBrain (Manjón and Coupé, 2016) did not differ significantly between the HC and ET groups (*p* = 0.2403, Table 1 and Supplementary Figure 1). Cerebellar lobules were segmented and volumetrically quantified using CERES (Romero et al., 2017) and analyzed using a multivariate general linear model with gender, age, and total brain volume as covariates. Total cerebellar volumes did not differ (right: *p* = 0.874 and left: *p* = 0.995, Figure 2A) between the groups, while volumes of lobules I-II (right: *p* < 0.001 and left: *p* < 0.001), left Crus II (*p* < 0.001), and left VIIB (*p* < 0.001) were lower in the ET group than in the HC group (Figures 2B,C and Table 2). The right lobule X was larger in the ET group (*p* < 0.001).

**TABLE 2:** Volumes of cerebellar lobules I–X in healthy controls (HCs) and in patients with essential tremor (ET) normalized to total brain tissue (reported as percentage).

		HC (N=55)		ET (N=55)	
Volume	Side	Average (%)	SD	Average (%)	SD
**Cerebellum**	R	5,435	0,439	5,398	0,479
	L	5,397	0,422	5,347	0,480
**I-II**	**R**	0,006*	**0,002**	0,004*	**0,001**
	**L**	0,005*	**0,002**	0,004*	**0,001**
**III**	R	0,058	0,011	0,062	0,013
	L	0,057	0,012	0,062	0,015
**IV**	R	0,183	0,027	0,189	0,034
	L	0,191	0,030	0,200	0,036
**V**	R	0,343	0,054	0,344	0,054
	L	0,353	0,052	0,350	0,050
**VI**	R	0,769	0,116	0,737	0,114
	L	0,750	0,088	0,719	0,100
**Crus I**	R	1,125	0,156	1,131	0,141
	L	1,101	0,151	1,107	0,157
**Crus II**	R	0,677	0,097	0,612	0,107
	**L**	0,666*	**0,089**	0,583*	**0,095**
**VIIB**	R	0,394	0,056	0,373	0,054
	**L**	0,394*	**0,054**	0,356*	**0,051**
**VIIIA**	R	0,508	0,066	0,479	0,088
	L	0,514	0,070	0,485	0,082
**VIIIB**	R	0,351	0,050	0,345	0,047
	L	0,350	0,052	0,359	0,045
**IX**	R	0,313	0,060	0,315	0,059
	L	0,302	0,059	0,301	0,065
**X**	**R**	0,055*	**0,008**	0,062*	**0,007**
	L	0,054	0,008	0,057	0,008

A multivariate general linear model was applied to the cerebellum and the 12 lobules (right and left sides) with gender, age, and total brain volume as covariates. The significance level p < 0.05 was adjusted for multiple testing using the Benjamini-Hochberg method. Significance denoted by * (bold). R: right and L: left.

## Discussion

Structural MRI-derived cerebellar volumes of a large sample of ET patients eligible for DBS were compared with those of matched HCs. Among ET patients, subtle volume reductions were observed in bilateral lobules I-II, left Crus II, and left VIIB. The volume of the right lobule X was increased.

The observations from the present study are partly concordant with those of previous studies. A lobule-based analysis demonstrated reduced gray matter volumes of VI and VIIA in a pooled cohort of 39 patients with cerebellar and classic ET (Shin et al., 2016). Another study used the spatially unbiased infratentorial segmentation pipeline and reported reduced gray matter densities in the left lobules I-IV, V, VIIB, VIIIA, IX, and X; right lobules V and IX; and in vermal regions in a subgroup of ET patients with head or jaw tremor (Dyke et al., 2017). Laterality of volumetric changes has been reported in cerebellar gray matter volume (Quattrone et al., 2008). The reduced volumes of Crus II and VIIB in the left cerebellum may be related to technical or biological aspects, which varied between the ET and HC cohorts. Handedness is an example of the latter, although the implications on cerebellar gray matter volumes remain questioned (Ocklenburg et al., 2016).

Interestingly, our results did not show pronounced volumetric reductions within the first (I-VI) and second (VIIIA-VIIIB) cerebellar motor representations (Buckner et al., 2011; Guell et al., 2018) among ET patients. Instead, the findings are consistent with a recent meta-analysis that challenged the hypothesis of distinct structural cerebellar atrophy patterns in ET and indicated that study size and heterogeneity might explain the diverse VBM findings reported previously (Luo et al., 2019). An alternative explanation for the reduced volumes in the present investigation might be the difference in MRI sites and scanner protocols, which influence the absolute volumetric measurements (Shinohara et al., 2017).

Histopathological examinations of ET patients demonstrated features suggesting neurodegeneration such as altered axon morphology and orientation of Purkinje cell bodies, reduced cell densities, and loss of dendritic spines (Louis, 2016). However, such changes may not necessarily cause volumetric differences observable on structural imaging. A recent resting-state functional MRI (fMRI) investigation indicated reduced cerebellar connectivity in ET, characterized by eigenvector centrality (Mueller et al., 2017). Additionally, evidence regarding the cerebellar involvement in ET has been obtained from fMRI investigations of ET patients wherein DBS affected cerebello-cerebral networks in task-dependent as well as task-independent manners (Awad et al., 2019). In addition, diffusion tensor imaging of patients with ET and Parkinson’s disease demonstrated increased radial diffusivity in the superior, medial, and inferior cerebellar peduncles in ET, suggesting white matter involvement (Juttukonda et al., 2018).

The present study is limited by the use of MRI data from different sources, retrieved over a long period (2006–2015). However, similar comparisons using MRI scans from the IXI repository have previously been applied to case series (Corral-Juan et al., 2018). To reduce the technical interference introduced by different T1-weighted MRI parameters, volumes were normalized to total brain tissue, which did not differ significantly between the HC and ET groups. Although the CERES and volBrain pipelines are fully automated, differential segmentations based on MRI scan parameters and image quality may affect the results (Manjón and Coupé, 2016; Romero et al., 2017). The present study did not perform corrections for disease severity, ET subtype, hand dominance, and relevant comorbidities such as vascular disease and alcohol consumption. Patient selection was based on ET severity and only the patients eligible for DBS were included, although some previous studies have supported (Bagepally et al., 2012) and others have opposed (Quattrone et al., 2008) a relationship between disease severity and structural gray matter changes.

## Conclusion

A cohort of 55 patients with ET demonstrated subtle signs of a decrease in cerebellar lobule volumes. These findings do not support the hypothesis of localized atrophy in the cerebellar motor areas in ET. Prospective investigations using structural or functional neuroimaging are needed to confirm these findings and to further elucidate the pathophysiology of ET to provide insights into diagnostic and therapeutic approaches.

## Data Availability Statement

The raw data supporting the conclusions of this article will be made available by the authors, without undue reservation, to any qualified researcher.

## Ethics Statement

The studies involving human participants were reviewed and approved by the Umeå Regional Ethical Review Board, Umeå, Sweden. Written informed consent for participation was not required for this study in accordance with the national legislation and the institutional requirements.

## Author Contributions

RÅ, AA, and AF conceived and planned the study. PB provided the data. RÅ and AA performed the data analysis. PB and AF interpreted the data. RÅ prepared the manuscript with support from AA, PB, and AF. AF supervised the study. All authors approved the final manuscript.

## Conflict of Interest

PB is a consultant for Abbott, Medtronic, and Boston Scientific. He is a shareholder in Mithridaticum AB. The remaining authors declare that the research was conducted in the absence of any commercial or financial relationships that could be construed as a potential conflict of interest.

## Supplementary Material

Supplementary Figure 1 Violin plots of total brain volumes for healthy controls (HC) and patients with essential tremor (ET), determined using the volBrain pipeline (Manjón and Coupé, 2016). The bars represent medians and the 25th and 75th percentiles. The average values and statistics are presented in Table 1.

## References

[B1] Awad A. Blomstedt P. Westling G. Eriksson J. (2019). Deep brain stimulation in the caudal zona incerta modulates the sensorimotor cerebello-cerebral circuit in essential tremor. *Neuroimage* 209:116511. 10.1016/j.neuroimage.2019.116511 31901420

[B2] Bagepally B. S. Bhatt M. D. Chandran V. Saini J. Bharath R. D. Vasudev M. K. (2012). Decrease in cerebral and cerebellar gray matter in essential tremor: a Voxel-Based Morphometric analysis under 3T MRI. *J. Neuroimag.* 22 275–278. 10.1111/j.1552-6569.2011.00598.x 21447032

[B3] Barbe M. T. Reker P. Hamacher S. Franklin J. Kraus D. Dembek T. A. (2018). DBS of the PSA and the VIM in essential tremor. *Neurology* 91:e543. 10.1212/WNL.0000000000005956 29970404

[B4] Benito-León J. (2014). Essential tremor: a neurodegenerative disease? *Tremor Other Hyperkinet. Mov. (N.Y.)* 4:252. 10.7916/D8765CG0 25120943 PMC4107287

[B5] Benito-Leon J. Alvarez-Linera J. Hernandez-Tamames J. A. Alonso-Navarro H. Jimenez-Jimenez F. J. Louis E. D. (2009). Brain structural changes in essential tremor: voxel-based morphometry at 3-Tesla. *J. Neurol. Sci.* 287 138–142. 10.1016/j.jns.2009.08.037 19717167

[B6] Benito-Leon J. Louis E. D. Bermejo-Pareja F. Neurological Disorders in Central Spain Study Group. (2006). Population-based case-control study of cognitive function in essential tremor. *Neurology* 66 69–74. 10.1212/01.wnl.0000192393.05850.ec 16401849

[B7] Benjamini Y. Hochberg Y. (1995). Controlling the false discovery rate: a practical and powerful approach to multiple testing. *J. R. Stat. Soc. Series B* 57 289–300. 10.1111/j.2517-6161.1995.tb02031.x

[B8] Bhalsing K. S. Upadhyay N. Kumar K. J. Saini J. Yadav R. Gupta A. K. (2014). Association between cortical volume loss and cognitive impairments in essential tremor. *Eur. J. Neurol.* 21 874–883. 10.1111/ene.12399 24612409

[B9] Buckner R. L. Krienen F. M. Castellanos A. Diaz J. C. Yeo B. T. (2011). The organization of the human cerebellum estimated by intrinsic functional connectivity. *J. Neurophysiol.* 106 2322–2345. 10.1152/jn.00339.2011 21795627 PMC3214121

[B10] Buijink A. W. van der Stouwe A. M. Broersma M. Sharifi S. Groot P. F. Speelman J. D. (2015). Motor network disruption in essential tremor: a functional and effective connectivity study. *Brain* 138 2934–2947. 10.1093/brain/awv225 26248468

[B11] Corral-Juan M. Serrano-Munuera C. Rábano A. Cota-González D. Segarra-Roca A. Ispierto L. (2018). Clinical, genetic and neuropathological characterization of spinocerebellar ataxia type 37. *Brain* 141 1981–1997. 10.1093/brain/awy137 29939198

[B12] Daniels C. Peller M. Wolff S. Alfke K. Witt K. Gaser C. (2006). Voxel-based morphometry shows no decreases in cerebellar gray matter volume in essential tremor. *Neurology* 67:1452. 10.1212/01.wnl.0000240130.94408.99 17060572

[B13] Dupuis M. J. M. Evrard F. L. A. Jacquerye P. G. Picard G. R. Lermen O. G. (2010). Disappearance of essential tremor after stroke. *Movement Disord.* 25 2884–2887. 10.1002/mds.23328 20836089

[B14] Dyke J. P. Cameron E. Hernandez N. Dydak U. Louis E. D. (2017). Gray matter density loss in essential tremor: a lobule by lobule analysis of the cerebellum. *Cerebellum Ataxias* 4:10. 10.1186/s40673-017-0069-3 28680651 PMC5494891

[B15] Elias W. J. Lipsman N. Ondo W. G. Ghanouni P. Kim Y. G. Lee W. (2016). A randomized trial of focused ultrasound thalamotomy for essential tremor. *N. Engl. J. Med.* 375 730–739. 10.1056/NEJMoa1600159 27557301

[B16] Fang W. Lv F. Luo T. Cheng O. Liao W. Sheng K. (2013). Abnormal regional homogeneity in patients with essential tremor revealed by resting-state functional MRI. *PLoS One* 8:e69199. 10.1371/journal.pone.0069199 23869236 PMC3711903

[B17] Fytagoridis A. Sandvik U. Aström M. Bergenheim T. Blomstedt P. (2012). Long term follow-up of deep brain stimulation of the caudal zona incerta for essential tremor. *J. Neurol. Neurosurg. Psychiatry* 83 258–262. 10.1136/jnnp-2011-300765 22205676

[B18] Gallea C. Popa T. García-Lorenzo D. Valabregue R. Legrand A.-P. Marais L. (2015). Intrinsic signature of essential tremor in the cerebello-frontal network. *Brain* 138 2920–2933. 10.1093/brain/awv171 26115677 PMC4747645

[B19] Guell X. Gabrieli J. D. E. Schmahmann J. D. (2018). Triple representation of language, working memory, social and emotion processing in the cerebellum: convergent evidence from task and seed-based resting-state fMRI analyses in a single large cohort. *Neuroimage* 172 437–449. 10.1016/j.neuroimage.2018.01.082 29408539 PMC5910233

[B20] Hallett M. (2014). Tremor: Pathophysiology. *Parkinsonism Relat. Disord.* 20 S118–S122. 10.1016/S1353-8020(13)70029-4 24262161

[B21] Juttukonda M. R. Franco G. Englot D. J. Lin Y.-C. Petersen K. J. Trujillo P. (2018). White matter differences between essential tremor and Parkinson disease. *Neurology* 92 e30–e39. 10.1212/WNL.0000000000006694 30504432 PMC6336163

[B22] Li X. Morgan P. S. Ashburner J. Smith J. Rorden C. (2016). The first step for neuroimaging data analysis: DICOM to NIfTI conversion. *J. Neurosci. Methods* 264 47–56. 10.1016/j.jneumeth.2016.03.001 26945974

[B23] Louis E. D. (2016). Essential tremor: a common disorder of purkinje neurons? *Neuroscientist* 22 108–118. 10.1177/1073858415590351 26044399 PMC5467972

[B24] Louis E. D. Benito-Leon J. Bermejo-Pareja F. Neurological Disorders in Central Spain Study Group. (2007). Self-reported depression and anti-depressant medication use in essential tremor: cross-sectional and prospective analyses in a population-based study. *Eur. J. Neurol.* 14 1138–1146. 10.1111/j.1468-1331.2007.01923.x 17708753

[B25] Louis E. D. Benito-Leon J. Ottman R. Bermejo-Pareja F. Neurological Disorders in Central Spain Study Group. (2007). A population-based study of mortality in essential tremor. *Neurology* 69 1982–1989. 10.1212/01.wnl.0000279339.87987.d7 18025392

[B26] Luo R. Pan P. Xu Y. Chen L. (2019). No reliable gray matter changes in essential tremor. *Neurol. Sci.* 40 2051–2063. 10.1007/s10072-019-03933-0 31115799

[B27] Manjón J. V. Coupé P. (2016). volBrain: an online MRI Brain volumetry system. *Front. Neuroinform.* 10:30. 10.3389/fninf.2016.00030 27512372 PMC4961698

[B28] Mueller K. Jech R. Hoskovcová M. Ulmanová O. Urgošík D. Vymazal J. (2017). General and selective brain connectivity alterations in essential tremor: A resting state fMRI study. *Neuroimage Clin.* 16 468–476. 10.1016/j.nicl.2017.06.004 28913163 PMC5587870

[B29] Nicoletti V. Cecchi P. Frosini D. Pesaresi I. Fabbri S. Diciotti S. (2015). Morphometric and functional MRI changes in essential tremor with and without resting tremor. *J. Neurol.* 262 719–728. 10.1007/s00415-014-7626-y 25572161

[B30] Ocklenburg S. Friedrich P. Güntürkün O. Genç E. (2016). Voxel-wise grey matter asymmetry analysis in left- and right-handers. *Neurosci. Lett.* 633 210–214. 10.1016/j.neulet.2016.09.046 27687715

[B31] Pietracupa S. Bologna M. Bharti K. Pasqua G. Tommasin S. Elifani F. (2019). White matter rather than gray matter damage characterizes essential tremor. *Eur. Radiol.* 29 6634–6642. 10.1007/s00330-019-06267-9 31139970

[B32] Quattrone A. Cerasa A. Messina D. Nicoletti G. Hagberg G. E. Lemieux L. (2008). Essential head tremor is associated with cerebellar vermis atrophy: a volumetric and Voxel-Based Morphometry MR Imaging Study. *Am. J. Neuroradiol.* 29:1692. 10.3174/ajnr.A1190 18653686 PMC8118768

[B33] Rajput A. H. Adler C. H. Shill H. A. Rajput A. (2012). Essential tremor is not a neurodegenerative disease. *Neurodegenerative Dis. Manage.* 2 259–268. 10.2217/nmt.12.23 23105950 PMC3478956

[B34] Romero J. E. Coupé P. Giraud R. Ta V.-T. Fonov V. Park M. T. M. (2017). CERES: a new cerebellum lobule segmentation method. *NeuroImage* 147 916–924. 10.1016/j.neuroimage.2016.11.003 27833012

[B35] Shin H. Lee D.-K. Lee J.-M. Huh Y.-E. Youn J. Louis E. D. (2016). Atrophy of the cerebellar vermis in essential tremor: segmental volumetric MRI analysis. *Cerebellum* 15 174–181. 10.1007/s12311-015-0682-8 26062905

[B36] Shinohara R. T. Oh J. Nair G. Calabresi P. A. Davatzikos C. Doshi J. (2017). Volumetric analysis from a harmonized multisite brain MRI study of a single subject with multiple sclerosis. *AJNR Am. J. Neuroradiol.* 38 1501–1509. 10.3174/ajnr.A5254 28642263 PMC5557658

[B37] Zesiewicz T. A. Chari A. Jahan I. Miller A. M. Sullivan K. L. (2010). Overview of essential tremor. *Neuropsychiat. Dis. Treatment* 6 401–408. 10.2147/ndt.s4795 20856604 PMC2938289

