# Supplementary figures and images for: Voxel-Based Morphometry of Cerebellar Lobules in Essential Tremor
Source: Front Aging Neurosci. 2021 Jun 10;13:667854. doi: 10.3389/fnagi.2021.667854 (PMC8222624; doi:10.3389/fnagi.2021.667854)

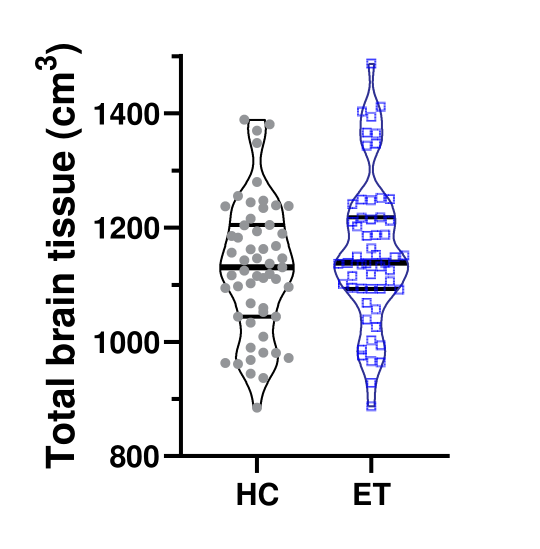

Supplement: Supplementary Figure 1 — Violin plots of total brain volumes for healthy controls (HC) and patients with essential tremor (ET), determined using the volBrain pipeline (Manjón and Coupé, 2016). The bars represent medians and the 25th and 75th percentiles. The average values and statistics are presented in Table 1. [file Image_1.tiff]
